# Supplementary material for: Cyclin F‐dependent degradation of E2F7 is critical for DNA repair and G2‐phase progression
Source: EMBO J. 2019 Sep 2;38(20):e101430. doi: 10.15252/embj.2018101430 (PMC6792010; doi:10.15252/embj.2018101430)
Supplement: Supplementary file 1 — Expanded View Figures PDF [file EMBJ-38-e101430-s001.pdf]

Expanded View Figures

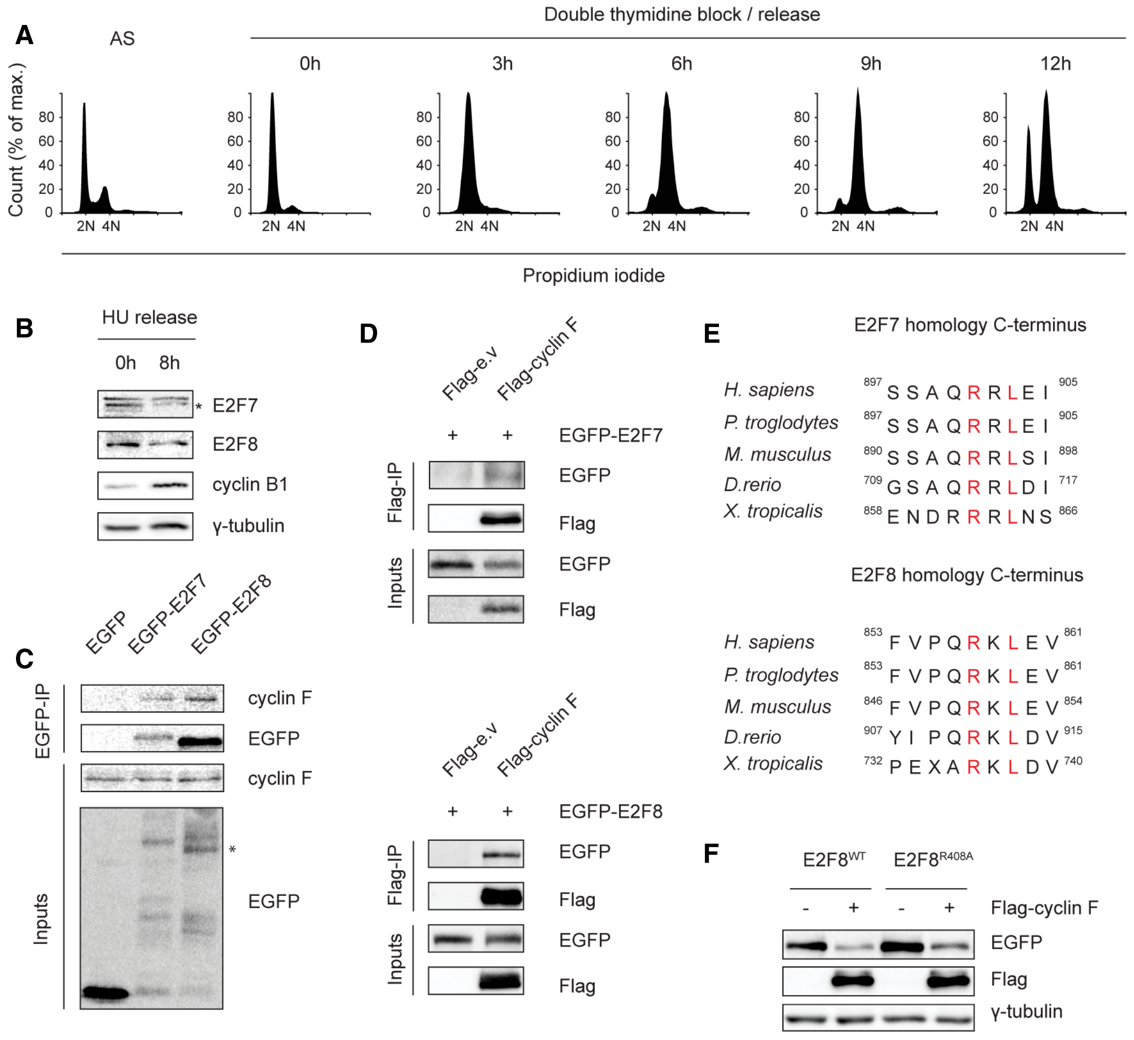

Figure EV1. Cyclin F binds to E2F7 and E2F8.

A Cell cycle analysis of the double thymidine block and release experiment shown in Fig 1A. HeLa cells were synchronized by double thymidine block and then released in fresh medium. Asynchronized (AS) and synchronized cells were harvested at the indicated time points for propidium iodide staining and flow cytometry analysis.

B E2F7 and E2F8 are degraded during G2/M phases. HeLa cells were treated with hydroxyurea (HU, 2 mM) for 16 h to arrest cells at G1/S border. Then, HU was removed and cells were released into fresh medium. Protein samples were harvested at the onset of release and 8 h after release. E2F7/8 levels were measured by immunoblotting. Protein expression of cyclin B1 was used as a marker for G2 or M cell cycle progression, and γ-tubulin was used as loading control. Asterisk indicates the specific band of E2F7 detection.

C Immunoprecipitation shows that cyclin F physically interacts with E2F7/8 *in vitro*. HEK293 cells were transiently transfected with either EGFP-tagged empty vector (EGFP), EGFP-tagged E2F7 or EGFP-tagged E2F8. MG132 was added to the cells 5 h before harvesting at 48 h post-transfection. Cells were harvested and lysed for immunoprecipitation using GFP resin. Asterisk indicates the E2F8-specific band.

D Reciprocal IP demonstrated the bindings between cyclin F and E2F7/8. HEK cells were transfected with Flag-tagged empty vector or cyclin F, together with GFP-tagged E2F7 or E2F8. MG132 was added to cells 5 h before harvesting, and co-IP was performed using Flag resin.

E Homology of atypical E2Fs at their C-terminus shows that the cyclin F-binding motifs are conserved among different species.

F Wild-type or R408A mutant of EGFP-tagged E2F8 was co-transfected with either empty vector or Flag-tagged cyclin F in HEK293 cells. Nocodazole was added to cells 8 h before harvest. Forty-eight hours after transfection, cells were collected and lysed for immunoblotting.

Source data are available online for this figure.

© 2019 The Authors

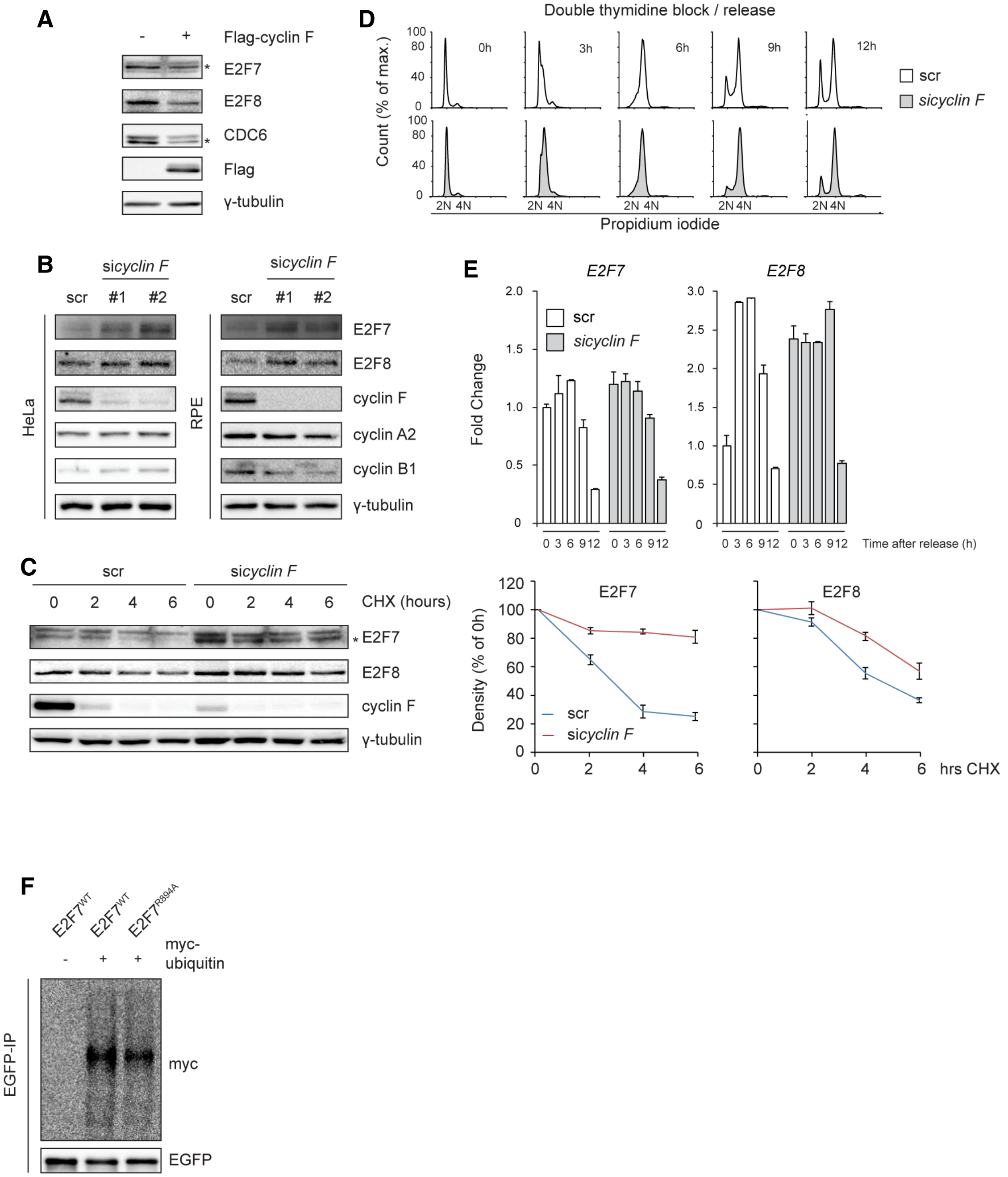

Figure EV2.

 © 2019 The Authors

Figure EV2. Cyclin F-dependent degradation of E2F7 and E2F8.

A Over expression of cyclin F downregulates endogenous E2F7 and E2F8. HEK cells were transiently transfected with Flag-tagged empty vector or cyclin F. Cells were harvested and lysed 48 h after transfection. Endogenous E2F7 and E2F8 were measured by immunoblotting. Detection of CDC6, a known cyclin F target, served as a positive control. Asterisk indicates the E2F7- and CDC6-specific band detection.

B Knockdown of cyclin F stabilized E2F7 and E2F8. HeLa and RPE cells were transfected with either scramble siRNA or individual cyclin F siRNA. Cells were harvested at 48 h post-transfection. Protein levels of E2F7/8 were analyzed by immunoblotting.

C The knockdown of cyclin F increases the half-life of E2F7/8. HeLa cells were transfected with scrambled siRNA (scr) or cyclin F siRNA (*sicyclin F*). Twenty-four hours after transfection, HeLa cells were treated with cycloheximide (CHX). Cells were harvested at the indicated time point after CHX treatment. Asterisk indicates the specific detection of endogenous E2F7. Quantifications (right panels) were performed based on two independent experiments. Error bars represent mean ± SEM.

D Cell cycle analysis of the double thymidine block and release of the experiment shown in Fig 3D. HeLa cells were transfected with siRNA against scrambled (scr) or cyclin F for 24 h. Then, cells were synchronized with double thymidine block and released into fresh medium. Samples were harvested at the indicated time points for propidium iodide staining and flow cytometry analysis.

E mRNA levels of E2F7 and E2F8 were not affected by knockdown of cyclin F. qPCR was preformed to analyze the double thymidine block and release of the experiment shown in Fig 3D. Bar and error bars represent mean ± SEM, n = 3.

F $E2F7^{R894A}$ mutant has reduction of ubiquitination. HEK cells were transiently transfected with either empty vector or myc-tagged ubiquitin, together with either EGFP-tagged $E2F7^{WT}$ or $E2F7^{R894A}$. MG132 was added to cells 5 h before harvesting, and EGFP-IP was performed.

Source data are available online for this figure.

© 2019 The Authors

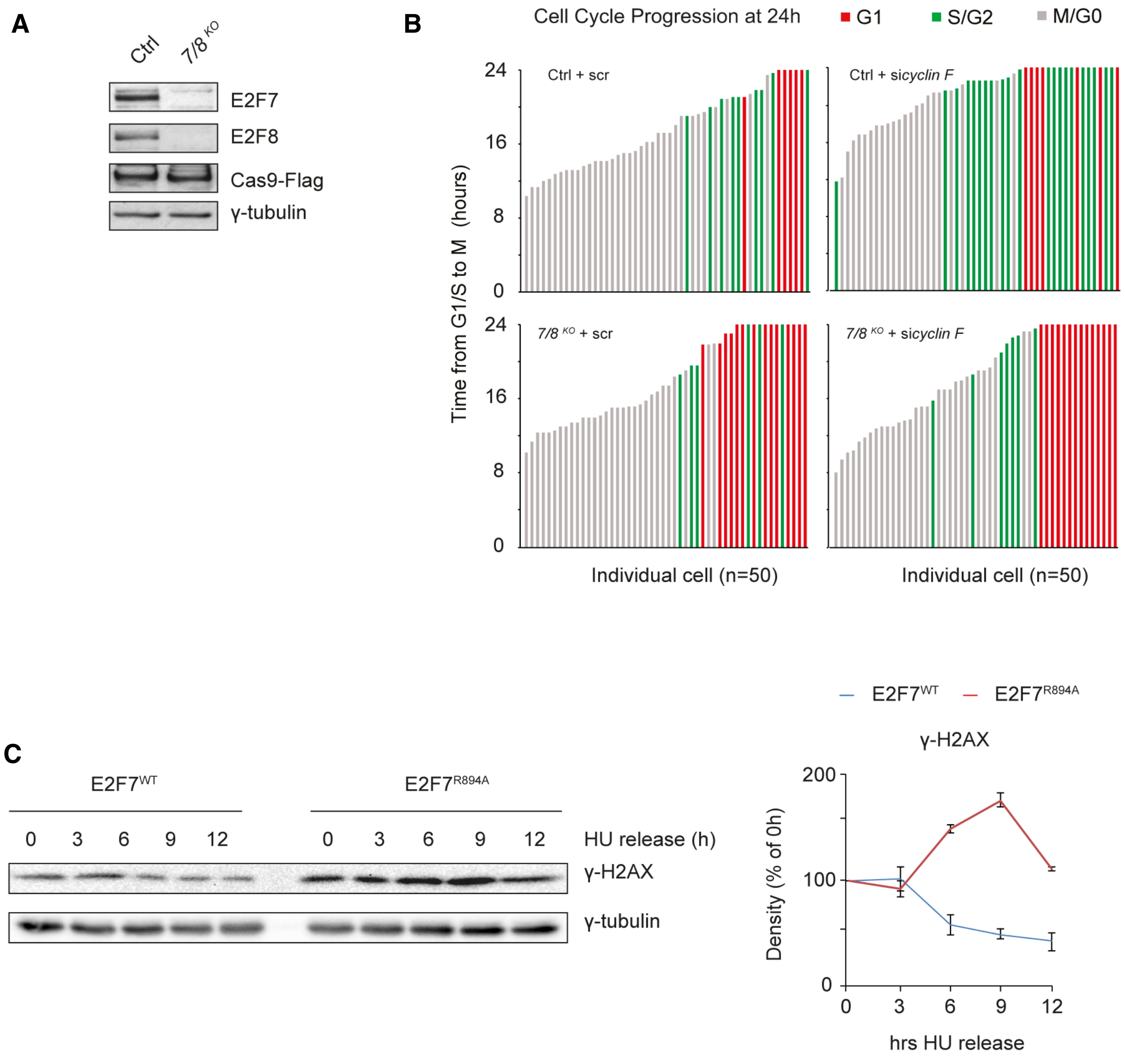

Figure EV3. Failure to degrade E2F7/8 caused cell cycle delay and DNA damage accumulation.

A Confirmation of efficient deletion of *E2F7* and *E2F8* in RPE-FUCCI cells utilizing CRISPR–CAS9 technology. Cells were transduced with Flag-tagged Cas9, and either a construct lacking sgRNA (Ctrl), or a construct containing sgRNA directed against *E2F7* and *E2F8* ($7/8^{KO}$). Protein levels were analyzed by immunoblotting.

B Depletion of cyclin F stalls the cell cycle at late S/G2. Each bar in the histogram shows the time that each individual of cell has spent from G1/S to Mitosis (correspond to Fig 4F). Red (G1), Green (S/G2) and Gray (M/G0) indicate the cell cycle stage of each cell after 24 h live cell imaging.

C Over expression of $E2F7^{R894A}$ mutant caused DNA damage. HeLa/TO cells were arrested with HU for 16 h and then released into fresh medium containing doxycycline. Protein samples were harvested every 3 h, and γ-H2AX was measured by immunoblotting (left). Quantifications were performed based on two independent experiments (right). Bars and error bars represent mean ± SEM.

Source data are available online for this figure.

 © 2019 The Authors

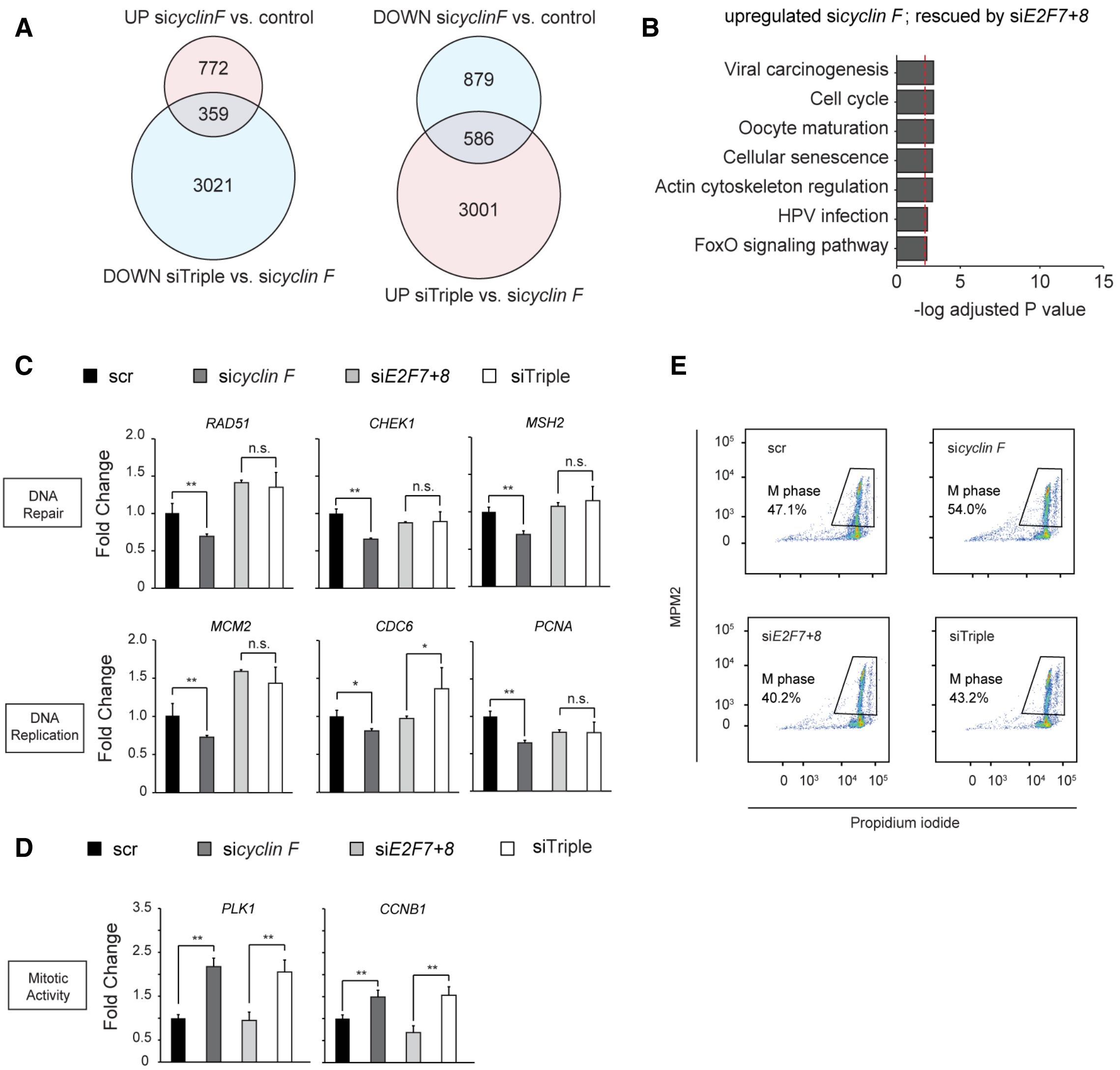

Figure EV4. Cyclin F controls transcription of DNA repair via E2F7/8.

A Venn diagrams of differentially expressed transcripts in RNA-sequencing analysis of nocodazole-arrested HeLa cells depleted of cyclin F and/or E2F7/8 as indicated.

B KEGG pathway analysis of genes upregulated by cyclin F knockdown and rescued by additional E2F7/8 depletion. Bars represent −log *P*-values, such that larger values mean stronger statistical significance. The cutoff *P*-value 0.05 is shown as a red dotted line.

C qPCR assay showing the expression of atypical E2F target genes that are involved in DNA damage and repair. RPE cells were transfected for 48 h with siRNA as indicated. Sixteen hours before harvesting, cells were treated with nocodazole. Data represent averages ± SEM (n = 3); *$P < 0.05$ or **$P < 0.01$ (Student's t-test). n.s.: not significant.

D qPCR showing the RNA expression of *PLK1* and *CCNB1*. HeLa cells were transfected for 48 h with siRNAs as indicated. Cells were incubated with nocodazole 16 h before harvesting. Data represent averages ± SEM (n = 3); **$P < 0.01$ (Student's t-test). n.s.: not significant.

E Phosphorylated MPM2 staining in flow cytometry demonstrated that mitotic activity was regulated in a cyclin F-dependent manner.

© 2019 The Authors

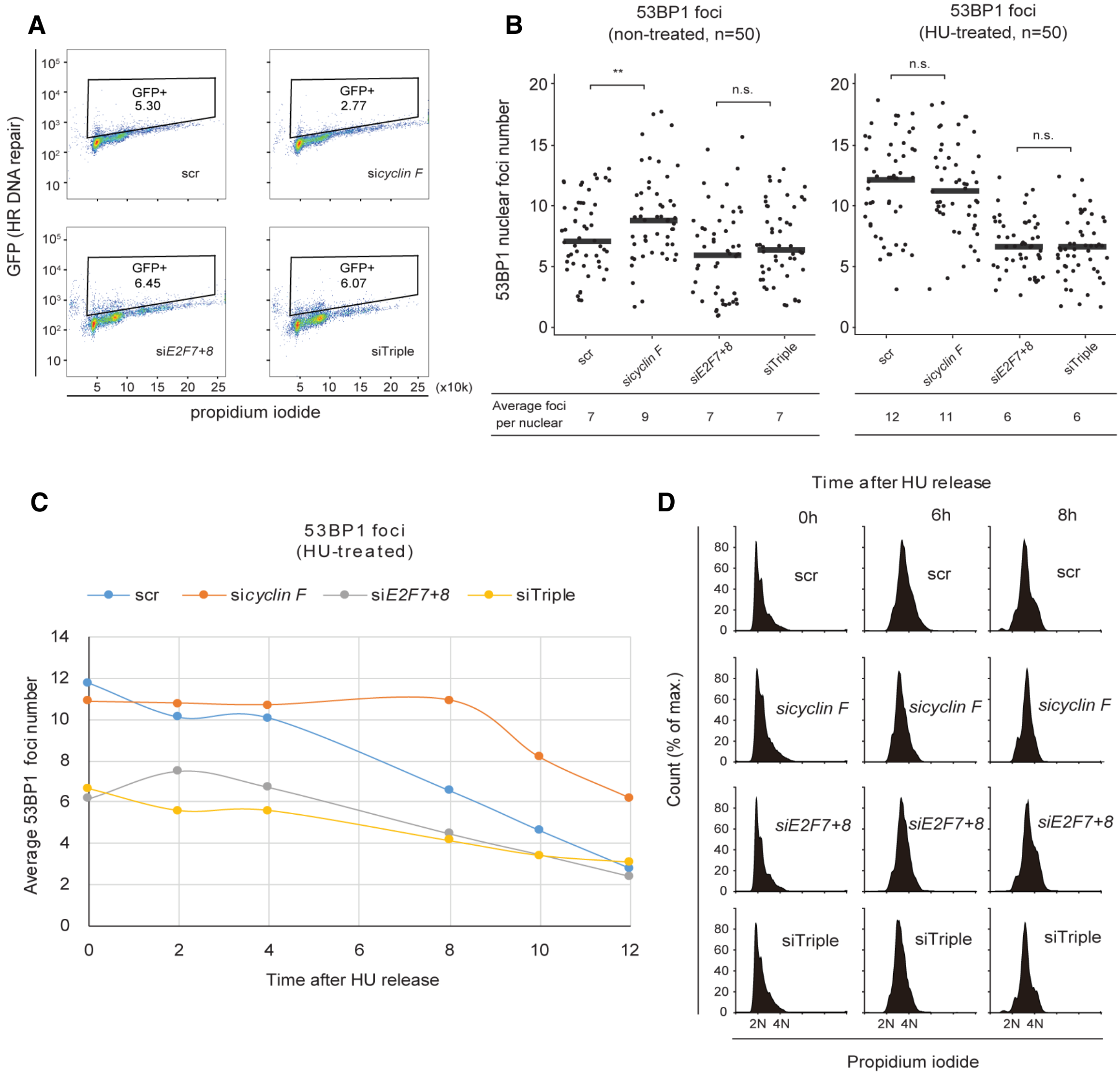

Figure EV5. Cyclin F-dependent degradation of E2F7/8 promotes DNA repair in G2.

A Loss of *cyclin F* induced E2F7/8-dependent homologous recombination deficiency. HeLa cells were transfected with siRNA as indicated. After 24 h, HeLa cells with stably transformed pDR-GFP were transfected with siRNA and harvested after 48 h for flow cytometry.

B Quantification of the 53BP1 foci at the beginning of live imaging (left: non-treated; right: HU-treated, 16 h). Dot plots show the 53BP1 foci number from each cell. The number of cells and the average foci per nucleus are shown in the table below. Black bars represent averages; $^{**}P < 0.01$ (Student's *t*-test) and n.s. (not significant).

C Dynamics of the number of 53BP1 foci per cell after HU release. At each time point after HU release, 50 random cells were picked and 53BP1 foci in each cell were counted.

D Flow cytometry showing the cell cycle progression of each condition at different time points after HU release.

 © 2019 The Authors
