# Supplementary figures and images for: Cyclin F‐dependent degradation of E2F7 is critical for DNA repair and G2‐phase progression
Source: EMBO J. 2019 Sep 2;38(20):e101430. doi: 10.15252/embj.2018101430 (PMC6792010; doi:10.15252/embj.2018101430)

**Fig EV1**

**B**

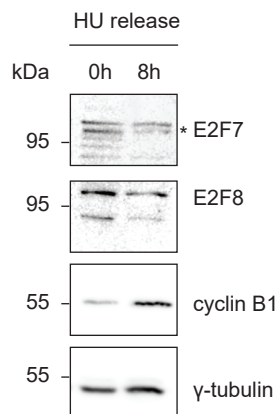

**C**

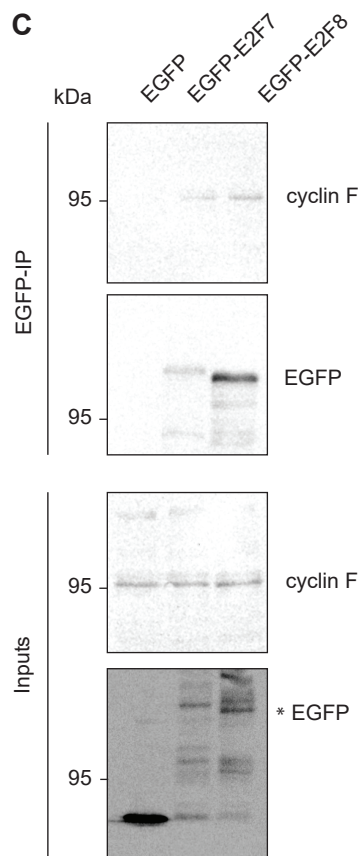

**D**

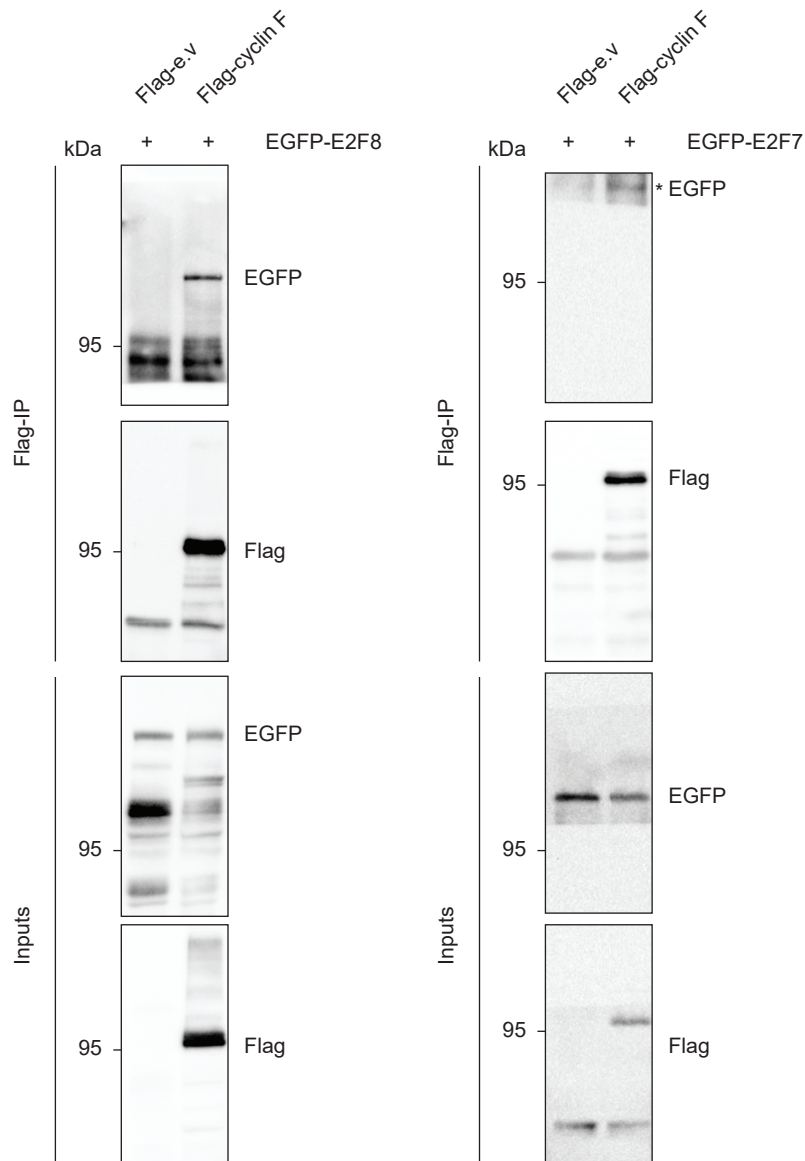

**F**

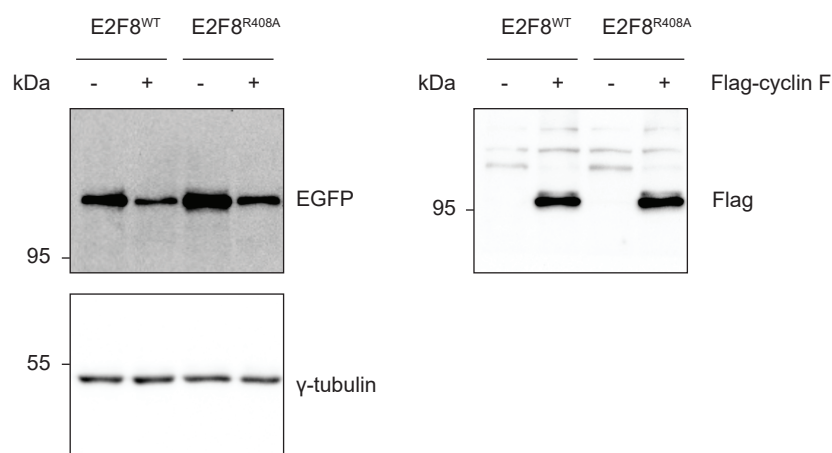

Supplement: Supplementary file 4 — Source Data for Expanded View and Appendix [file EMBJ-38-e101430-s009.zip › Source_Data_Figure_EV1.pdf]

**Fig EV2****A**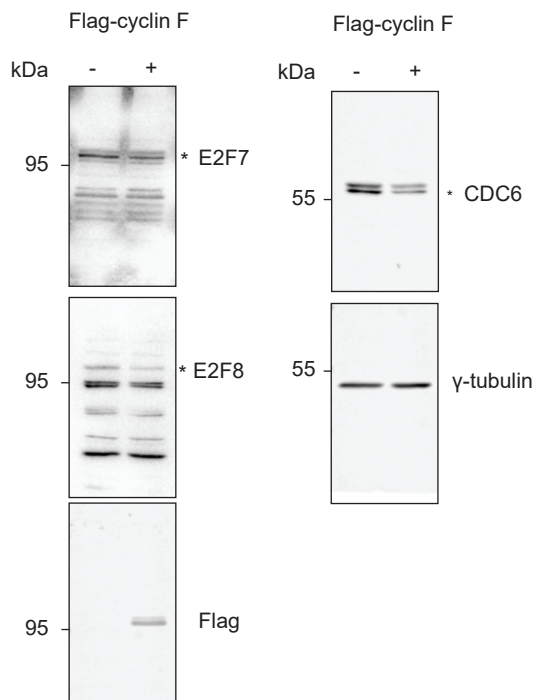**B**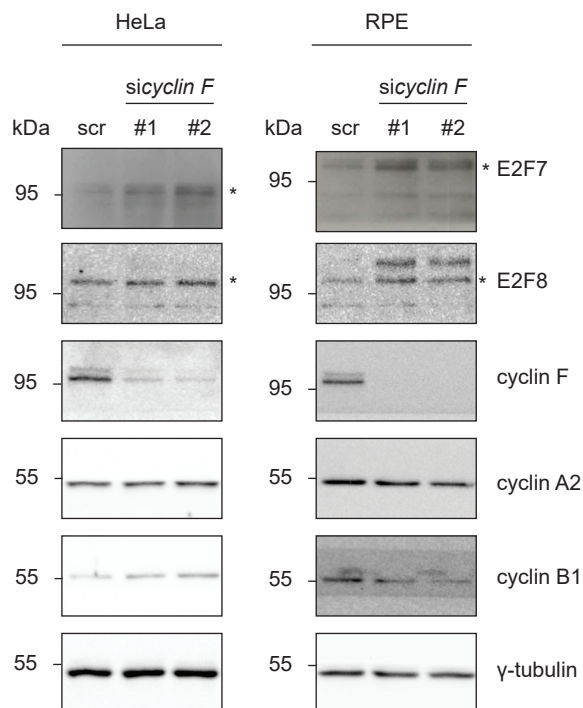**C**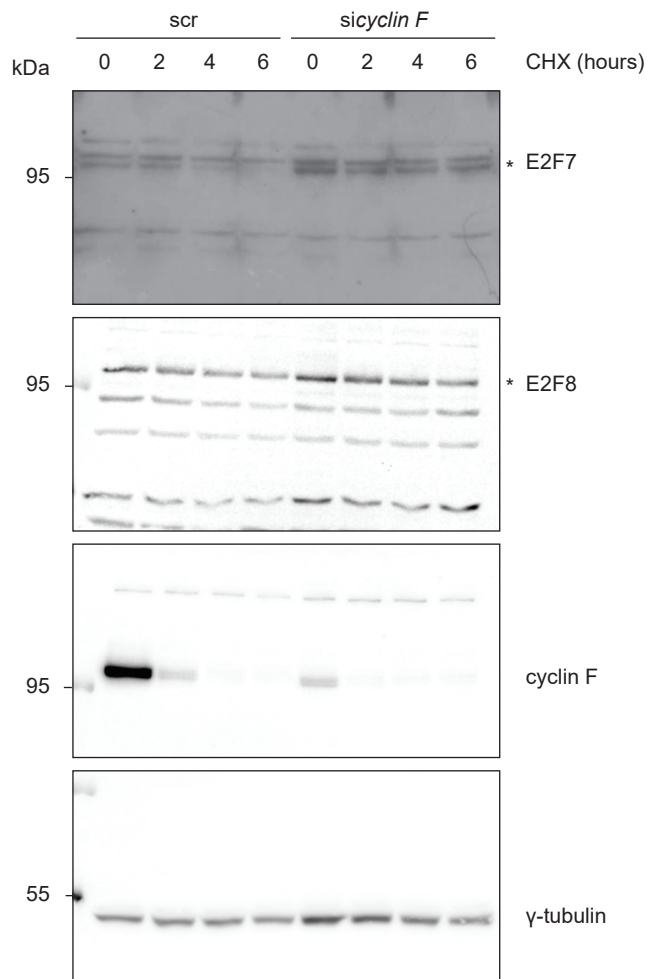**F**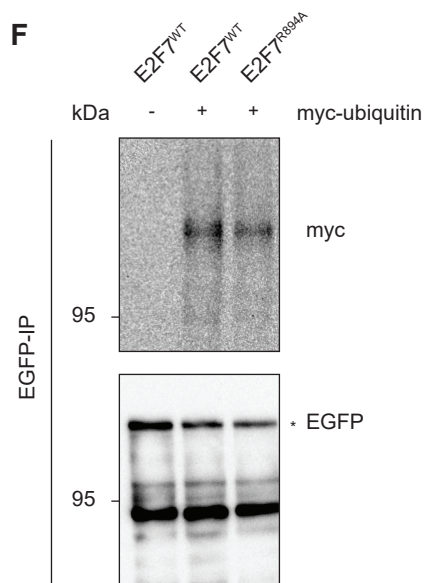

Supplement: Supplementary file 4 — Source Data for Expanded View and Appendix [file EMBJ-38-e101430-s009.zip › Source_Data_Figure_EV2.pdf]

Fig EV3

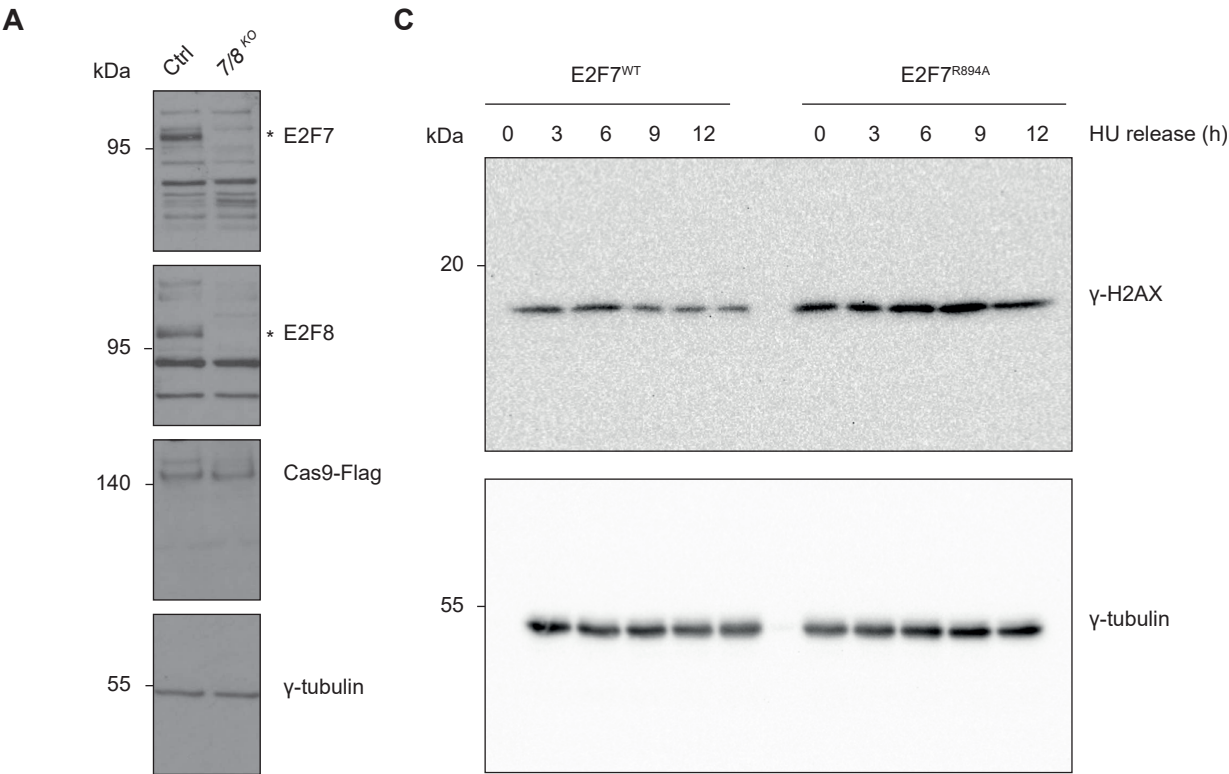

Supplement: Supplementary file 4 — Source Data for Expanded View and Appendix [file EMBJ-38-e101430-s009.zip › Source_Data_Figure_EV3.pdf]

Review Process File, Appendix Figure 1. SD.

A

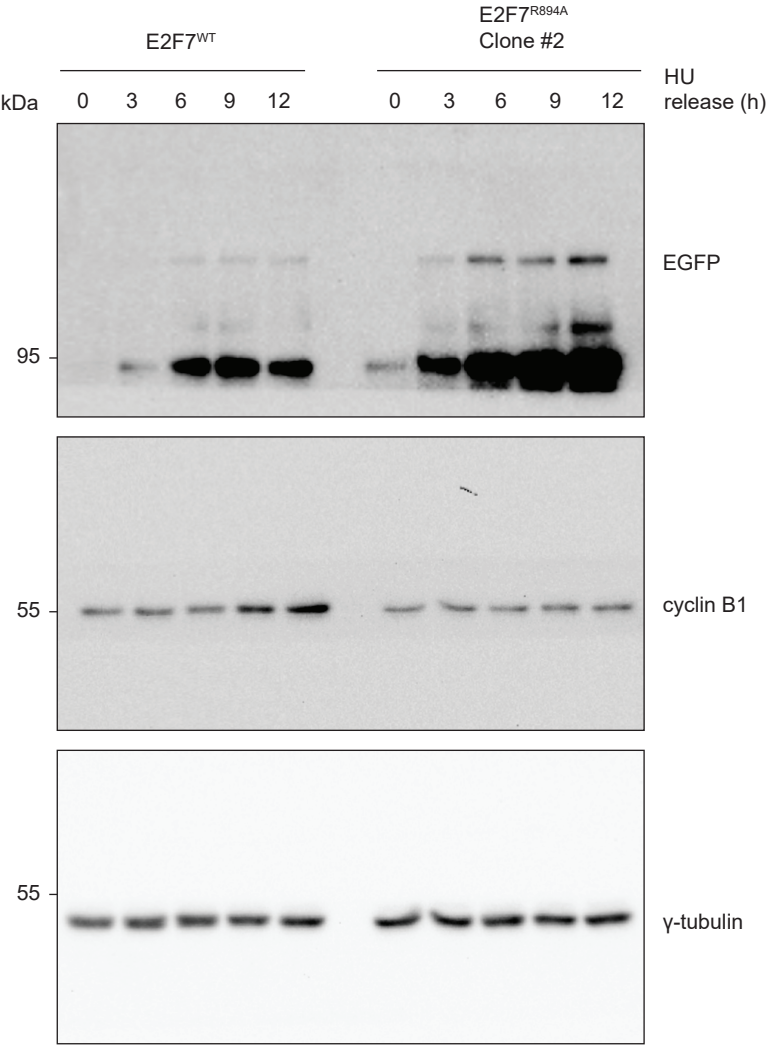

B

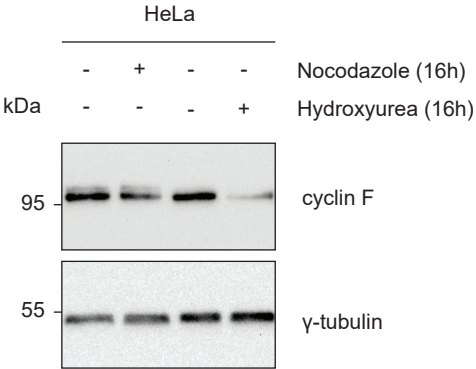

Supplement: Supplementary file 4 — Source Data for Expanded View and Appendix [file EMBJ-38-e101430-s009.zip › RPF_-_Appendix_Figure_1_SD.pdf]

**Fig 1****A**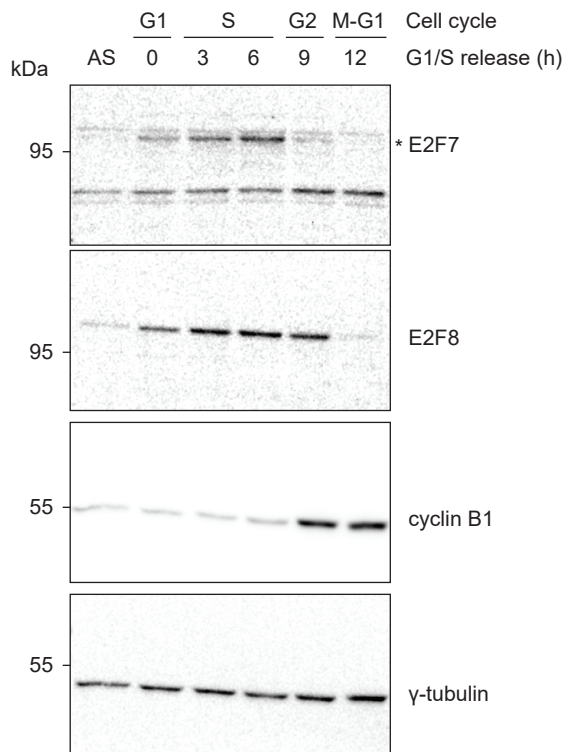**B**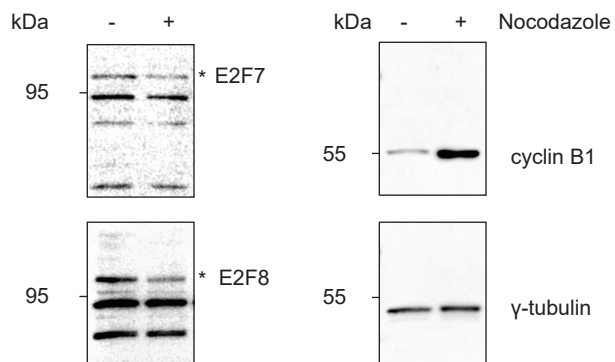**D**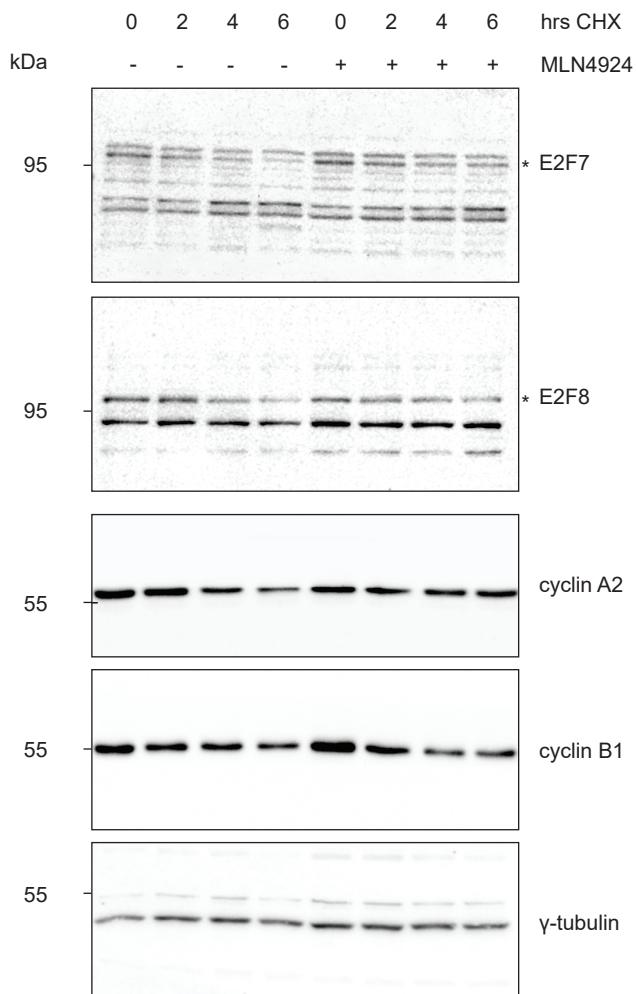**C**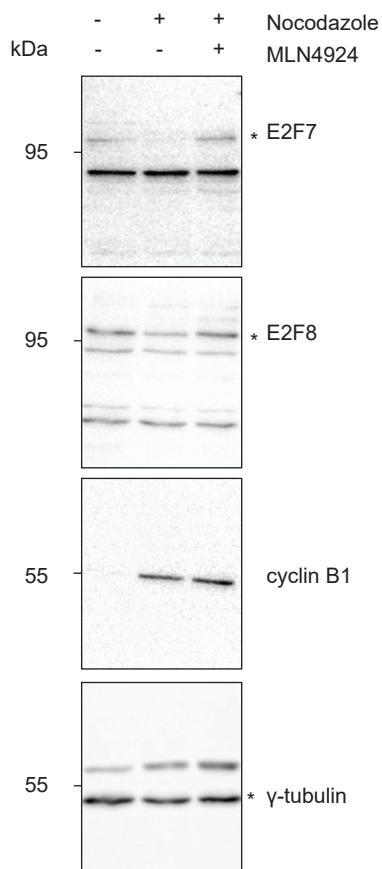

Supplement: Supplementary file 6 — Source Data for Figure 1 [file EMBJ-38-e101430-s004.pdf]

**Fig 2****B**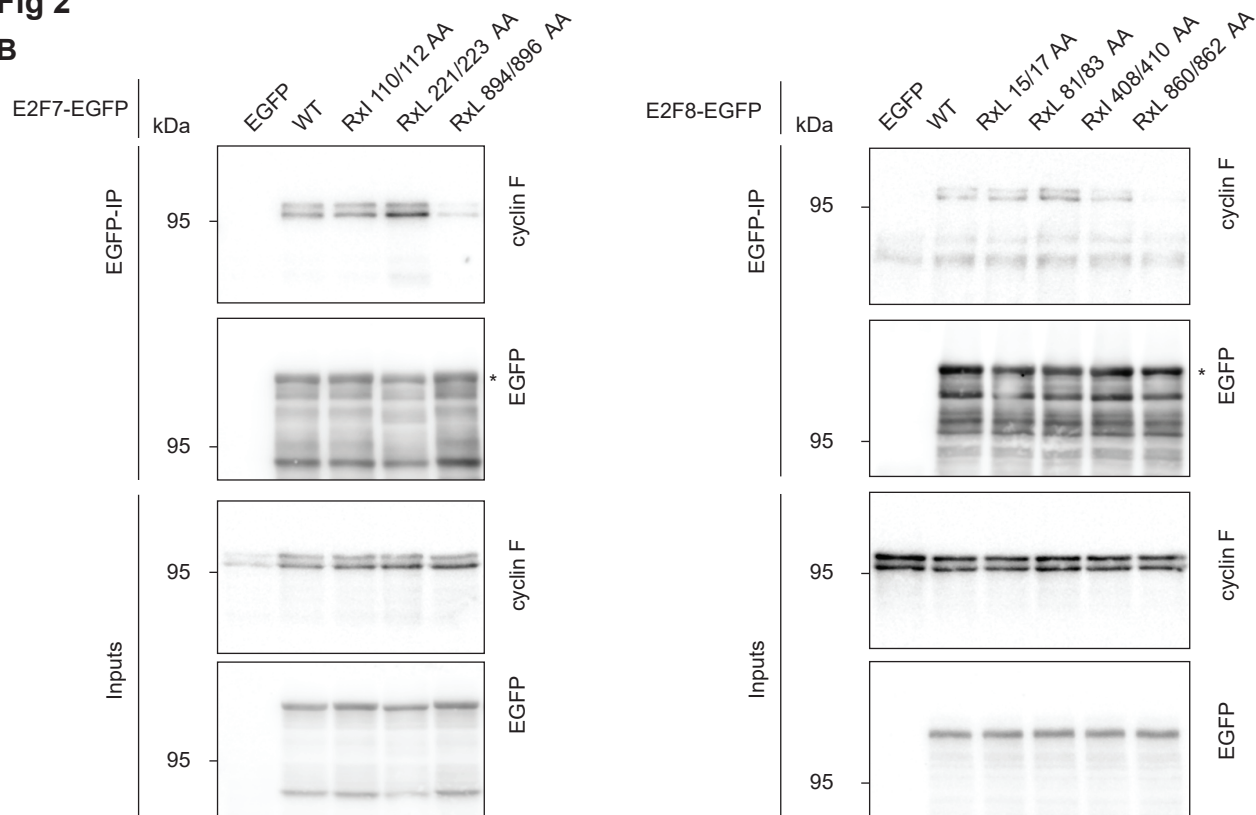**C**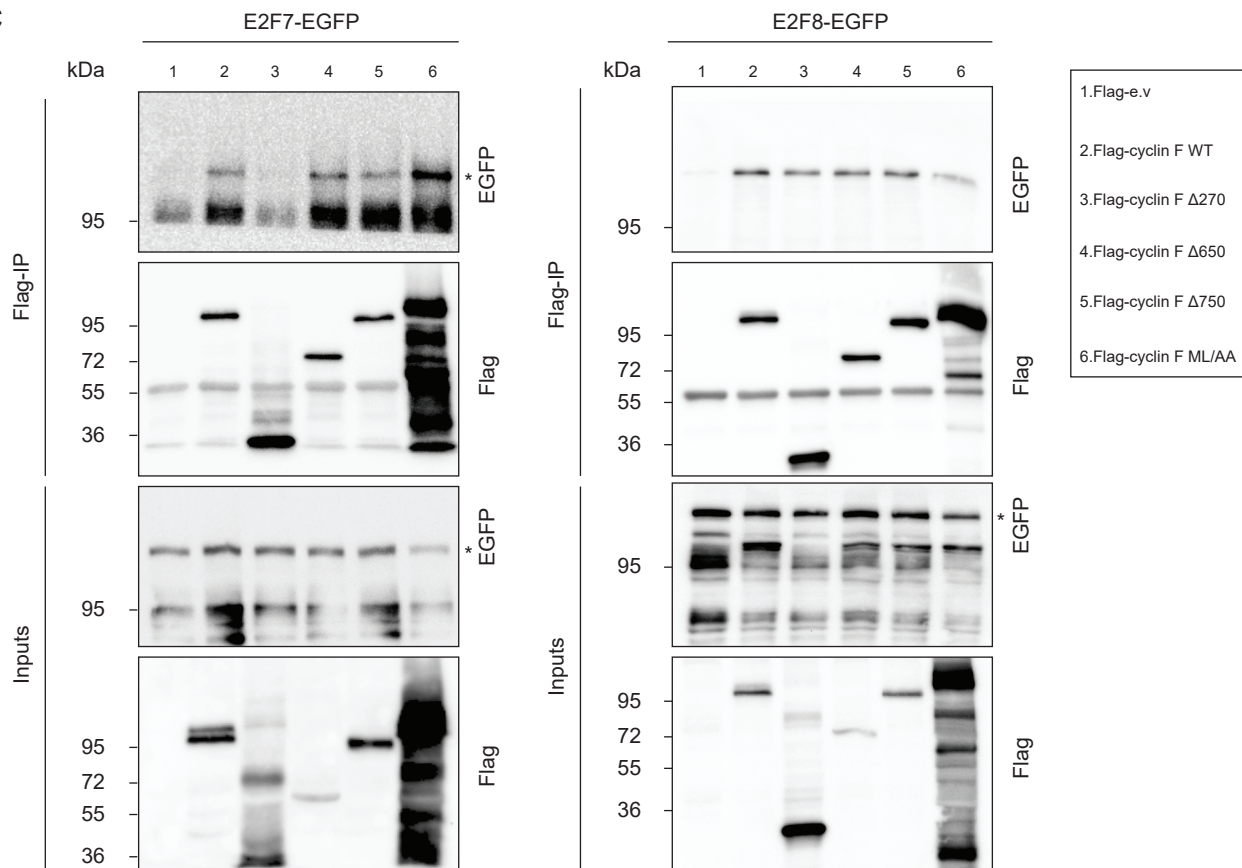

Supplement: Supplementary file 7 — Source Data for Figure 2 [file EMBJ-38-e101430-s005.pdf]

**Fig 3**

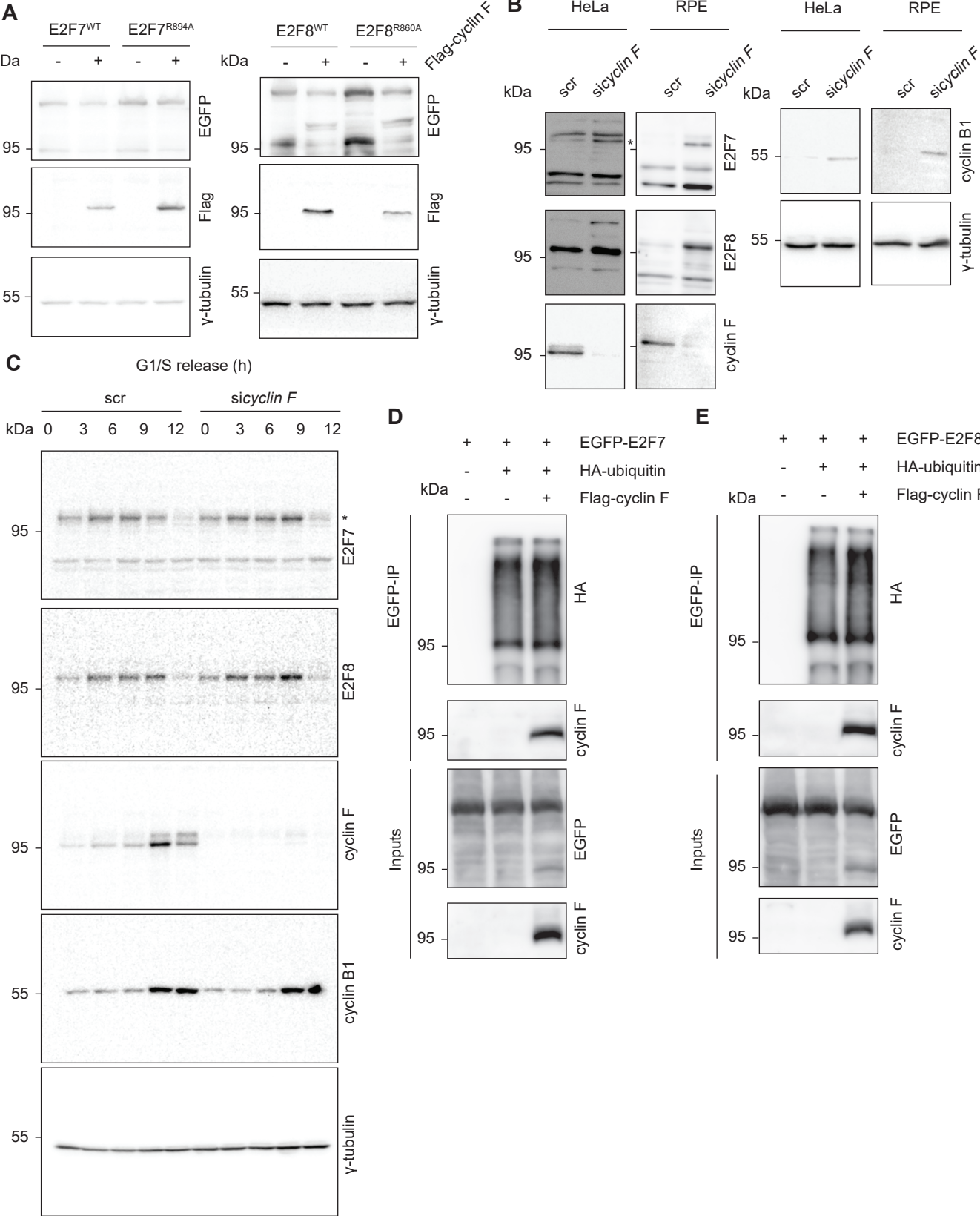

Supplement: Supplementary file 8 — Source Data for Figure 3 [file EMBJ-38-e101430-s006.pdf]

**Fig 5**

**A**

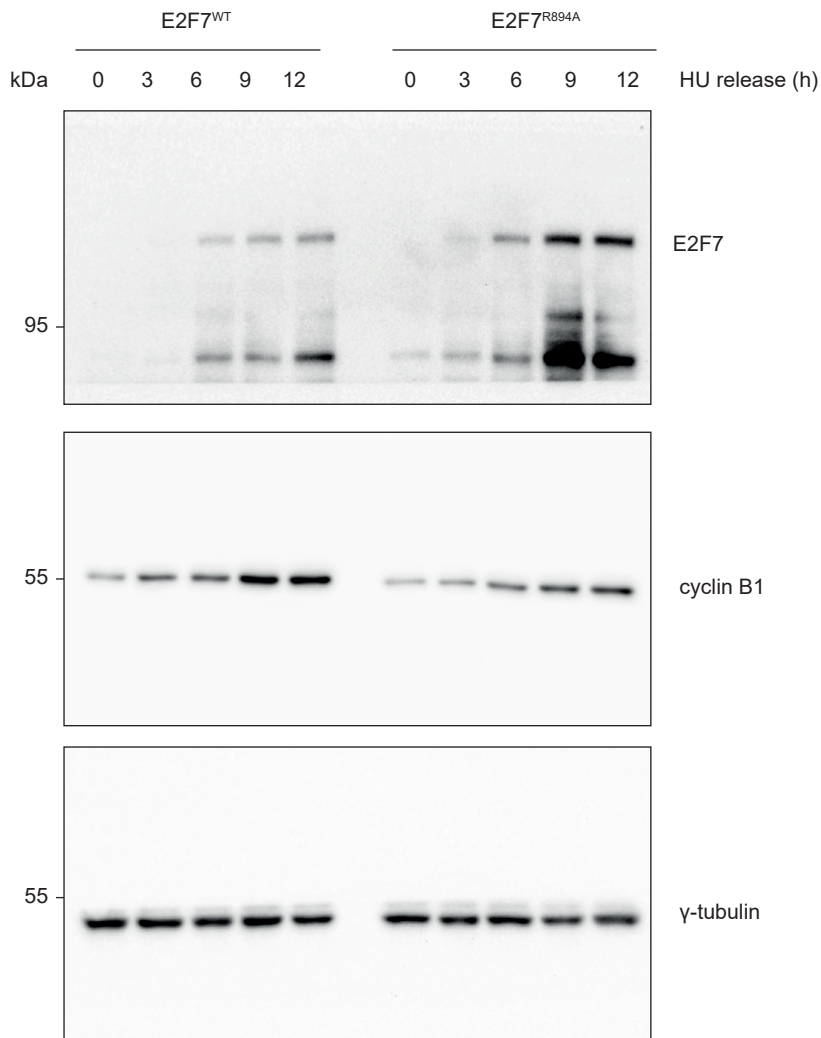

Supplement: Supplementary file 9 — Source Data for Figure 5 [file EMBJ-38-e101430-s007.pdf]

**Fig 6**

**D**

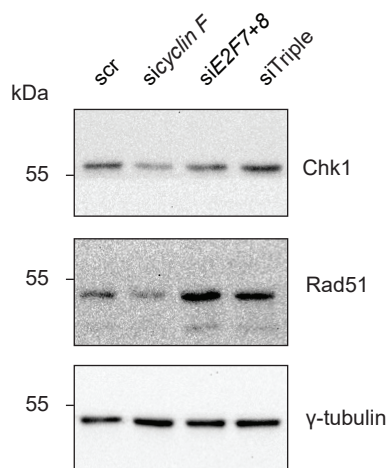

Supplement: Supplementary file 10 — Source Data for Figure 6 [file EMBJ-38-e101430-s008.pdf]
